# CT texture analysis of mediastinal lymphadenopathy: Combining with US-based elastographic parameter and discrimination between sarcoidosis and lymph node metastasis from small cell lung cancer

**DOI:** 10.1371/journal.pone.0243181

**Published:** 2020-12-02

**Authors:** Eriko Koda, Tsuneo Yamashiro, Rintaro Onoe, Hiroshi Handa, Shinya Azagami, Shoichiro Matsushita, Hayato Tomita, Takeo Inoue, Masamichi Mineshita

**Affiliations:** 1 Division of Respiratory Medicine, Department of Internal Medicine, St. Marianna University School of Medicine, Kawasaki, Kanagawa, Japan; 2 Department of Radiology, St. Marianna University School of Medicine, Kawasaki, Kanagawa, Japan; 3 Department of Diagnostic Radiology, Yokohama City University, Yokohama, Kanagawa, Japan; University of Texas MD Anderson Cancer Center, UNITED STATES

## Abstract

**Objectives:**

To investigate the potential of computed tomography (CT)-based texture analysis and elastographic data provided by endobronchial ultrasonography (EBUS) for differentiating the mediastinal lymphadenopathy by sarcoidosis and small cell lung cancer (SCLC) metastasis.

**Methods:**

Sixteen patients with sarcoidosis and 14 with SCLC were enrolled. On CT images showing the largest mediastinal lymph node, a fixed region of interest was drawn on the node, and texture features were automatically measured. Among the 30 patients, 19 (12 sarcoidosis and 7 SCLC) underwent endobronchial ultrasound transbronchial needle aspiration, and the fat-to-lesion strain ratio (FLR) was recorded. Texture features and FLRs were compared between the 2 patient groups. Logistic regression analysis was performed to evaluate the diagnostic accuracy of these measurements.

**Results:**

Of the 31 texture features, the differences between 11 texture features of CT ROIs in the patients with sarcoidosis versus patients with SCLC were significant. Among them, the grey-level run length matrix with high gray-level run emphasis (GLRLM-HGRE) showed the greatest difference (P<0.01). Differences between FLRs were significant (P<0.05). Logistic regression analysis together with receiver operating characteristic curve analysis demonstrated that the FLR combined with the GLRLM-HGRE showed a high diagnostic accuracy (100% sensitivity, 92% specificity, 0.988 area under the curve) for discriminating between sarcoidosis and SCLC.

**Conclusion:**

Texture analysis, particularly combined with the FLR, is useful for discriminating between mediastinal lymphadenopathy caused by sarcoidosis from that caused by metastasis from SCLC.

## Introduction

The causes of mediastinal lymphadenopathy are diverse, and include malignant diseases such as lung cancer and malignant lymphoma and benign diseases such as sarcoidosis and inflammation/infection. In daily clinical care for mediastinal lymphadenopathy, it is relatively common that no abnormal lesions are seen in the lung field, whereas multiple enlarged lymph nodes are observed in the mediastinum. For example, small cell lung cancer (SCLC) metastases and sarcoidosis are typical diseases that sometimes involve mediastinal lymph nodes without manifesting obvious lung lesions. In such cases, distinguishing benign from malignant diseases by computed tomography (CT) or even by ^18^F-fluorodeoxyglucose positron emission tomography/CT (FDG-PET/CT) is difficult [1, 2]. Texture analysis of chest CT data has been used for images of benign and malignant lung lesions and mediastinal lymphadenopathy associated with various diseases [3–10]. Although a limited number of published articles are available, the potential of CT-based texture analysis as a new imaging approach for distinguishing between benign and malignant lesions in the lung and mediastinum has gradually been recognized. However, to the best of our knowledge, no previous study has investigated whether or not CT-based texture analysis can be used to discriminate between the mediastinal lymphadenopathy of sarcoidosis and that of SCLC.

Endobronchial ultrasound transbronchial needle aspiration (EBUS-TBNA) is an important option for the histopathological evaluation of mediastinal lymphadenopathy. The technique of EBUS-TBNA has rapidly developed over recent years. During the process of EBUS-TBNA, the biopsied lymph node can be visualized on ultrasound imaging; and ultrasound elastography of the lymph node of interest has been investigated as a method for distinguishing between benign and malignant lymph nodes [11–14]. Although discriminating between benign and malignant lymph nodes by elastography alone has been difficult [15], it has been suggested that the fat-to-lesion strain ratio (FLR) determined by elastography is useful for differentiating between benign and malignant lesions outside of the mediastinum, particularly for breast cancer [16–18]. For lung cancer, Rozman et al compared the EBUS-based color-pattern diagnosis and the FLR for mediastinal lymphadenopathy and found that the FLR was more accurate than color-pattern diagnosis [19]. Together, the research findings on CT and EBUS elastography suggest that the results of the texture analysis of CT data and the FLR data provided by EBUS elastography can be used for discriminating between the mediastinal lymphadenopathy of sarcoidosis and that of SCLC. Furthermore, the combination of data obtained by CT and EBUS might provide high diagnostic accuracy.

Thus, the aim of this study was to investigate the potential of CT-based texture analysis and FLR data provided by EBUS elastography for differentiating the mediastinal lymphadenopathy of sarcoidosis from that of SCLC metastasis.

## Materials and methods

This retrospective study was approved by the Institutional Review Board of St. Marianna University School of Medicine. Patient consent for reviewing medical records was waived by the Board.

### Patients

We reviewed the medical records and chest CT scans of all the patients who underwent contrast-enhanced CT at our institution and were diagnosed with mediastinal lymphadenopathy caused by sarcoidosis or metastatic SCLC between January 2012 and May 2019. A total of 49 patients were initially identified. Nineteen patients with slice thicknesses that were not 0.5 mm-thickness on CT were excluded. A total of 30 patients (16 with sarcoidosis, 14 with SCLC) were finally enrolled in this study. The results from histopathological examinations of EBUS-TBNA of the mediastinal lymph nodes confirmed the diagnosis in all 16 patients with sarcoidosis and in 8 patients with SCLC. SCLC was confirmed from lung lesions or a metastatic cervical lymph node in the remaining 6 patients with SCLC, and not by aspirates of mediastinal lymph nodes, as follows: diagnosis was performed of a transbronchial lung biopsy (TBLB) specimen from the primary lung lesion in 3 patients, a CT-guided biopsy specimen of the primary lung lesion in 1 patient, an endobronchial biopsy specimen of the primary lesion in the proximal bronchus in 1 patient, and an ultrasonography-guided biopsy specimen of a metastatic cervical lymph node in 1 patient. The enlarged mediastinal lymph nodes of these 6 patients who did not undergo an EBUS-TBNA were clinically diagnosed as metastatic SCLC based on marked reductions in the size of the lymph nodes on follow-up CT after treatment initiation (chemotherapy and/or radiation therapy). The clinical characteristics of the patients are shown in Table 1.

**Table 1:** Clinical characteristics of study patients.

	Sarcoidosis (n = 16)		Small cell carcinoma (n = 14)	
Age (mean ± SD)	58 ± 14 years		73 ± 9 years	
Gender (male/female)	(11/5)		(11/3)	
Diagnostic method	EBUS-TBNA	16	EBUS-TBNA	8
			TBLB	3
			EBB	1
			US-guided biopsy	1
			CT-guided biopsy	1

**Abbreviations:** EBUS-TBNA = Endobronchial ultrasound transbronchial needle aspiration. TBLB = transbronchial lung biopsy. EBB = endobronchial biopsy. US = ultrasonography. CT = computed tomography

### CT scanning

All patients were scanned by one of 3 CT scanners (Aquilion, 64-row detector CT scanner; Aquilion ONE, 320-row detector; Aquilion PRIME, 80-row detector; Canon Medical Systems, Otawara, Tochigi, Japan). The scanning parameters were as follows: collimation, 0.5 mm; tube voltage, 120 kVp; tube current, automatic exposure control (AEC); gantry rotation time, 0.5 seconds; and beam pitch, 0.813 or 0.828. All images were reconstructed using an FC14 convolution kernel (for mediastinum) with a slice thickness of 0.5 mm. The imaging field of view ranged from 320 to 400 mm based on patient’s body habitus, with a matrix of 512 × 512. Contrast medium (1.5 mL/kg body weight) was injected over 60 seconds, and the patient was scanned 90 seconds later.

### Image analysis

**Fig 1:**
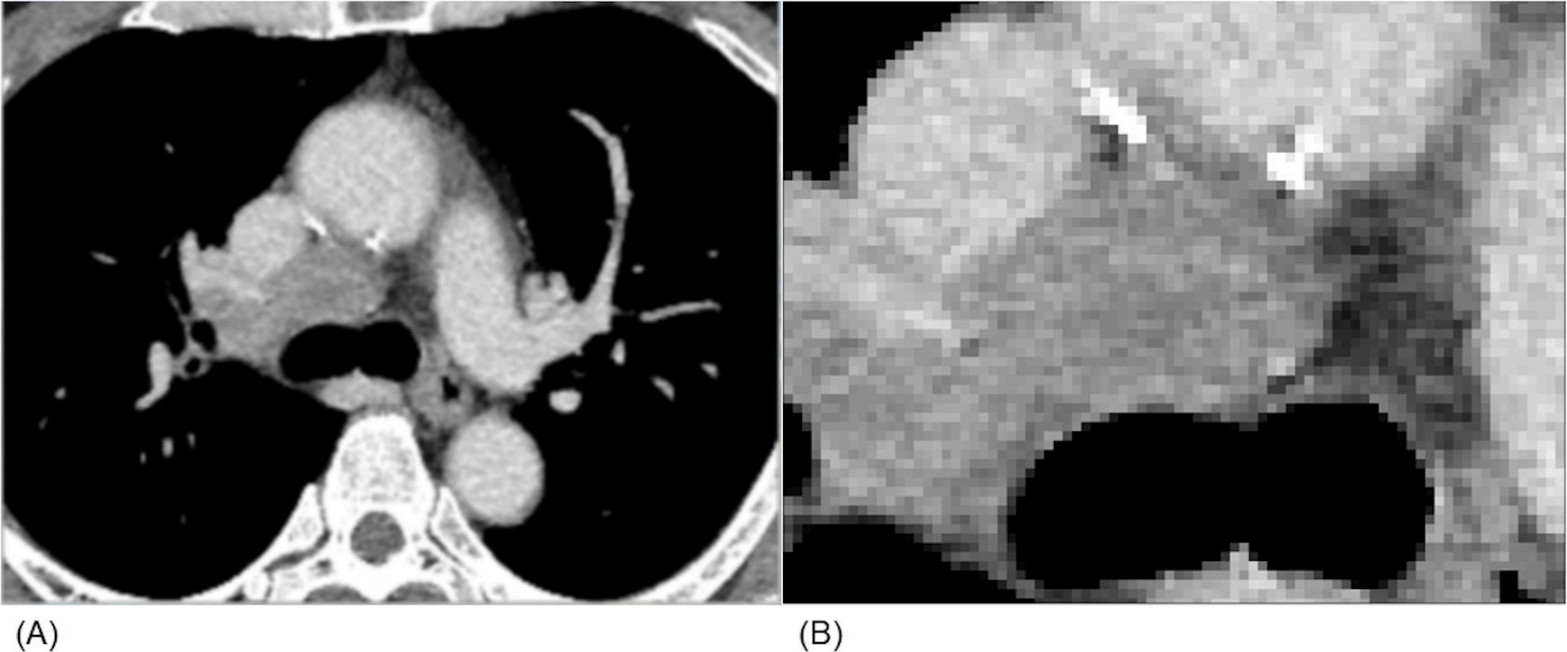
CT image of lymph node metastasis from small cell lung cancer. A large lymph node, which was proven by a transbronchial needle aspirate to be a lymph node metastasis of small cell lung cancer, is depicted on an enhanced CT image (A). Density and contrast enhancement of the node appears slightly heterogeneous; however, massive necrosis is not observed (B).

**Fig 2:**
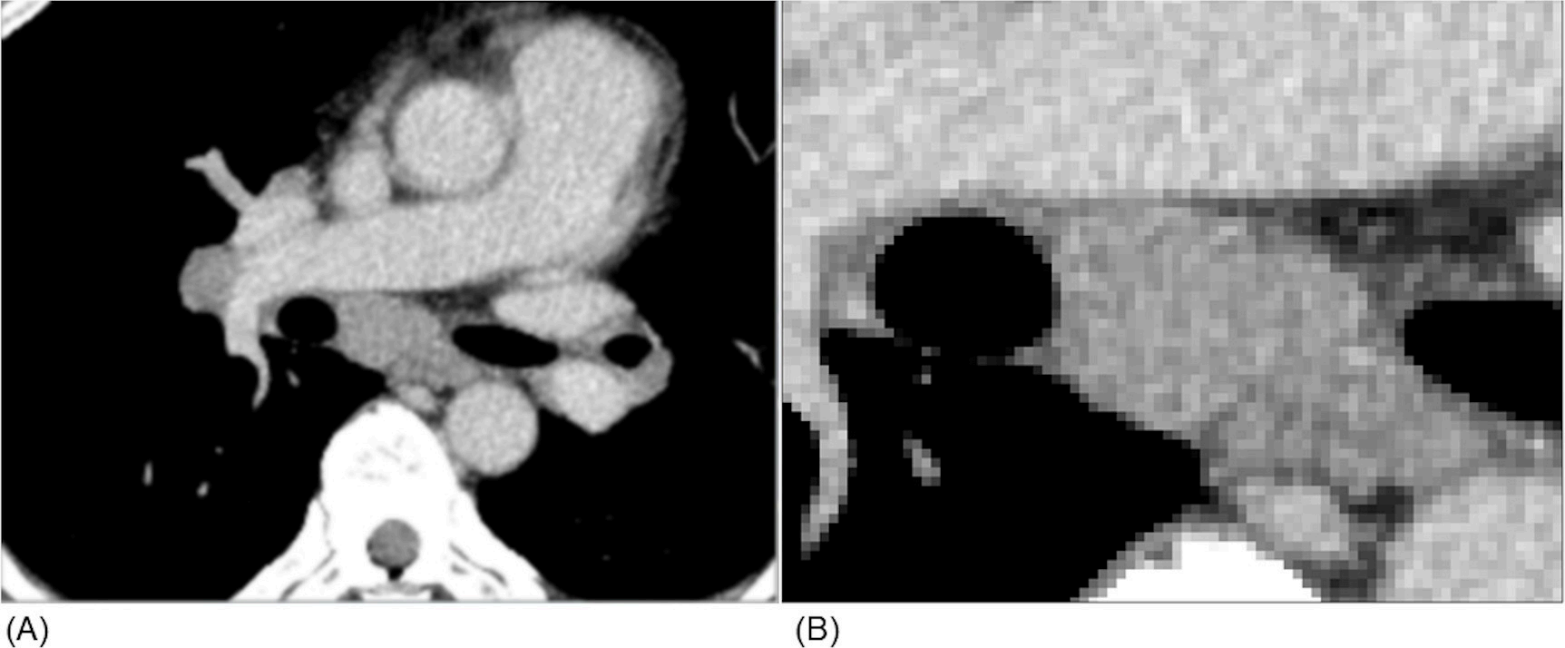
CT image of enlarged lymph nodes due to sarcoidosis. An enlarged mediastinal lymph node, as well as slightly enlarged hilar nodes, are shown on an enhanced CT image (A). The density of the mediastinal lymph node seems basically homogeneous (B); however, distinguishing enlargement due to sarcoidosis from that due to lymph node metastasis from small cell lung cancer on CT images only is difficult.

**Fig 3:**
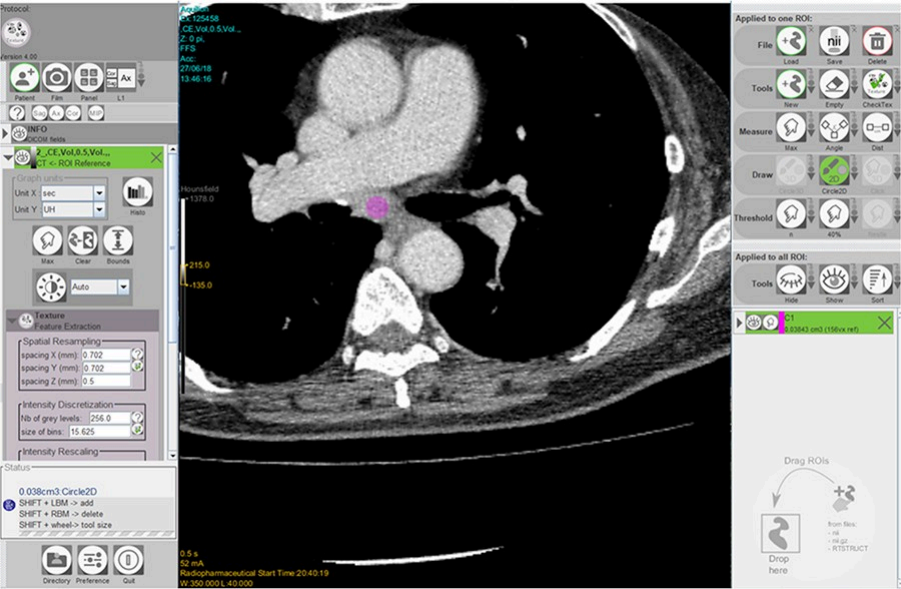
An example of texture analysis performed by the software. The open-source software (LIFEx) performed texture analysis of a 10-mm-diamter circular region of interest (colored pink), which was placed in the center of the targeted lymph node.

The CT-based texture analysis was performed on the enlarged mediastinal lymph nodes that were diagnosed as manifesting sarcoidosis or metastatic disease due to SCLC (Figs 1 and 2). For the 24 patients who underwent diagnostic EBUS-TBNA of their mediastinal lymph nodes, the biopsied lymph nodes were identified on CT scans and analyzed. For the remaining 6 SCLC patients who did not undergo EBUS-TBNA, texture analysis was performed on the largest of the enlarged mediastinal lymph nodes. A 10-mm-diameter region of interest (ROI) was placed on the center of the lymph node to be analyzed, by a single-blind observer who did not know the ultimate diagnosis of each patient. We assessed several texture features, using an open-source software module (LIFEx: http://www.lifexsoft.org) (Fig 3). Texture features were classified as follows: 5 histogram features, 7 gray level co-occurrence matrix (GLCM) features, 8 gray level run length matrix (GLRLM) features, 3 neighborhood gray level different matrix (NGLDM) features, and 8 gray level zone length matrix (GRZLM) features [20–22]. CT images were displayed on a LIFEx monitor with a window level of 40 Hounsfield Units (HU) and a window width of 350 HU.

### Bronchoscopy and elastography

The fat-to-lesion strain ratio (FLR) was recorded in 19 (12 with sarcoidosis and 7 with SCLC) of the 24 patients who underwent EBUS-TBNA. FLR was not recorded in 5 patients undergoing EBUS-TBNA, because the old EBUS system was not equipped with elastography technology.

EBUS-TBNA was performed by an ultrasonic puncture bronchoscope equipped with a linear, convex curved-array ultrasonic transducer (BF-UC260FW, Olympus, Tokyo, Japan). Mediastinal lymph node imaging was performed in the B mode of the ultrasonic puncture bronchoscope; elastography was performed to obtain the FLR, which is the ratio of the strain of the surrounding fat tissue to the strain of the lesion. To obtain the FLR value, a circular ROI was drawn on an ultrasound image of the targeted lymph node, and the hardness of the lymph node and that of the mediastinal fat tissue were compared. The size of the ROI varied, depending on the size of the targeted lymph node. Generally, hard lesions show elevated FLR values.

### Statistical analysis

Statistical analysis was performed by JMP Pro 14.1.0 software (SAS Institute, Cary, NC, USA). The Mann-Whitney U test was used to compare the values of CT-based texture features and FLR values of lymph nodes involved with sarcoidosis and those involved with SCLC. The value of P< 0.05 were considered statistically significant. Receiver operating characteristic (ROC) curve analysis and the area-under-the ROC (AUROC) curve values for some CT-based texture features and the FLR values were also determined in the assessment of diagnostic accuracy with regard to the differentiation between sarcoidosis and SCLC. Multinomial logistic regression analysis was performed to assess a combination of parameters.

## Results

### Differences between the CT texture features and the EBUS elastographic FLR values of the lymph nodes of patients with sarcoidosis versus patients with SCLC

**Fig 4:**
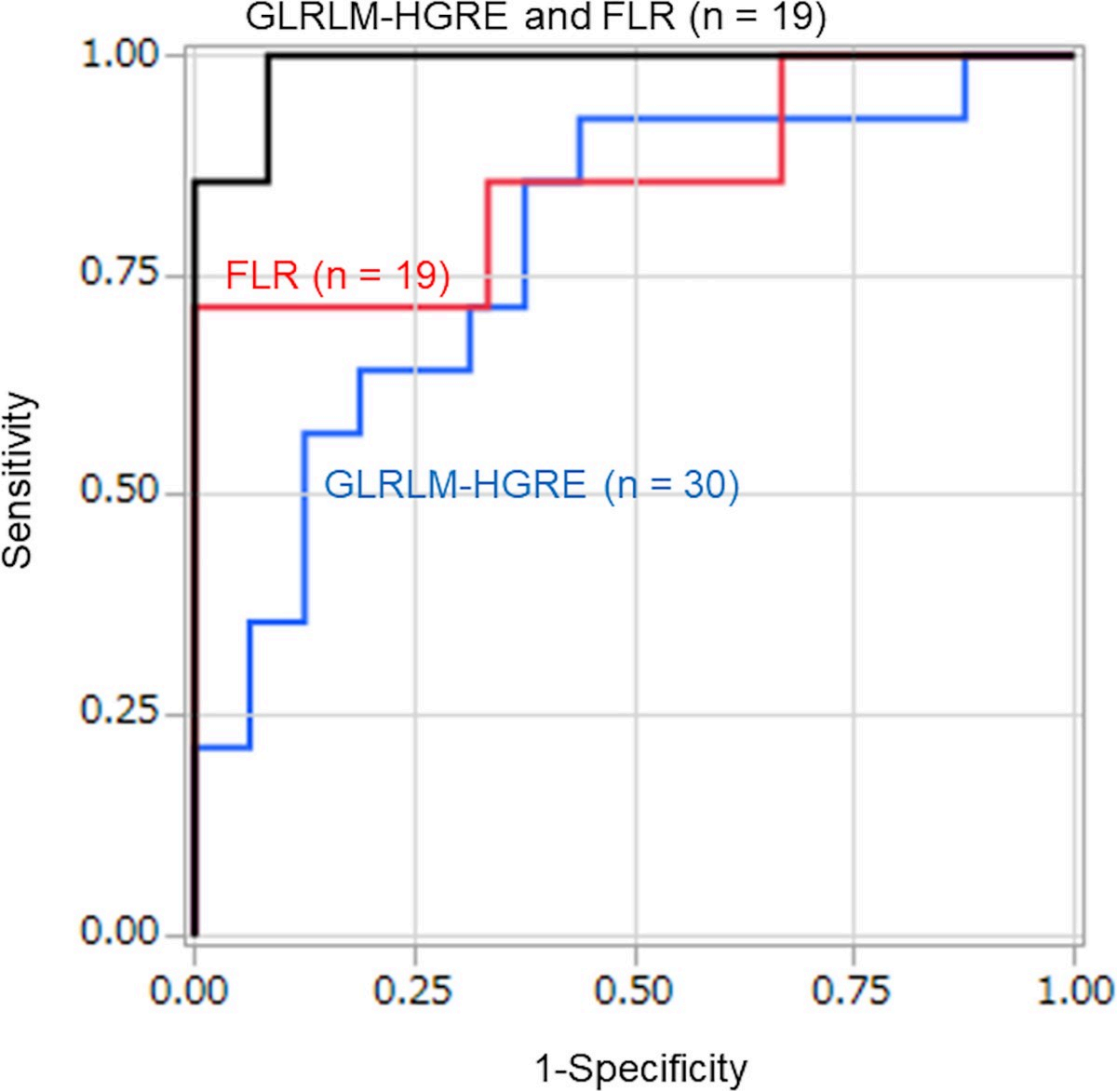
Receiver Operating Characteristic (ROC) curves of the values for CT-based texture features and US elastographic FLRs. ROC curves of the grey-level run length matrix (GLRLM)-high gray-level emphasis (HGRE) feature and the fat-to-lesion strain ratio (FLR), and the ROC of the combination of GLRLM-HGRE and FLR, which shows almost perfect diagnostic accuracy for distinguishing between lymphadenopathy due to sarcoidosis and that due to metastasis from small cell lung cancer. The area-under-the-curve values were as follows: GLRLM-HGRE alone, 0.781; FLR alone, 0.857; combination of GLRLM-HGRE plus FLR, 0.988.

Table 2 shows the differences between the imaging measurements in the patients with sarcoidosis versus those with SCLC. Among the 31 texture features analyzed in this study for the 30 patients, differences between 11 features in the sarcoidosis versus SCLC patients were significant. Among the features, the difference between the GLRLM with high gray-level-run emphasis (HGRE) features showed the greatest significance (P = 0.009). ROC curve analysis of GLRLM-HGRE, with a cut-off value of 4889, showed an AUROC of 0.781, and a sensitivity and specificity of 93% and 56%, respectively (Fig 4). The GLCM-contrast features demonstrated the second greatest significance (P = 0.016) with an AUROC of 0.759, and a sensitivity and specificity of 93% and 63% (cut-off value of 1.558), respectively.

**Table 2:** Radiomics features of lymph nodes affected by sarcoidosis versus the features of lymph nodes affected by small cell carcinoma.

Parameters		Sarcoidosis	Small cell carcinoma	P value
Histogram (n = 30)	Skewness	-0.31 ± 0.63	0.05 ± 0.30	N.S.
	Kurtosis	3.86 ± 1.87	3.60 ± 0.78	N.S.
	Entropy_log10	0.69 ± 0.09	0.75 ± 0.09	N.S.
	Entropy_log2	2.28 ± 0.30	2.48 ± 0.29	N.S.
	Energy	0.25 ± 0.04	0.22 ± 0.04	N.S.
GLCM (n = 30)	Homogeneity	0.632 ± 0.046	0.584 ± 0.063	0.03
	Energy	0.076 ± 0.025	0.057 ± 0.019	N.S.
	Contrast	1.647 ± 0.732	2.629 ± 1.501	0.016
	Correlation	0.470 ± 0.125	0.386 ± 0.098	N.S.
	Entropy_log10	1.27 ± 0.15	1.41 ± 0.16	0.048
	Entropy_log2	4.22 ± 0.50	4.67 ± 0.54	0.048
	Dissimilarity	0.927 ± 0.207	1.174 ± 0.342	0.018
GLRLM (n = 30)	SRE	0.756 ± 0.046	0.795 ± 0.052	N.S.
	LRE	2.88 ± 0.59	2.44 ± 0.51	0.048
	HGRE	4911.8 ± 131.5	4796.8 ± 125.8	0.009
	SRHGE	3713.3 ± 254.7	3815.4 ± 291.1	N.S.
	LRHGE	14159.4 ± 2930.5	11665.2 ± 2434.4	0.028
	GLNU	29.2 ± 3.4	27.9 ± 3.2	N.S.
	RLNU	71.5 ± 13.8	85.9 ± 20.7	N.S.
	RP	0.692 ± 0.052	0.739 ± 0.062	N.S.
NGLDM (n = 30)	Coarseness	0.042 ± 0.006	0.040 ± 0.005	N.S.
	Contrast	0.040 ± 0.010	0.047 ± 0.019	N.S.
	Busyness	873×10^−3^ ± 0.592	622×10^−3^ ± 0.259	N.S.
GLZLM (n = 30)	SZE	0.479 ± 0.118	0.551 ± 0.967	N.S.
	LZE	86.72 ± 62.58	46.56 ± 29.20	0.046
	HGZE	4896.0 ± 141.5	4803.4 ± 124.0	N.S.
	SZHGE	2340.8 ± 587.9	2651.8 ± 481.6	N.S.
	LZHGE	427349.3 ± 308783.4	222578.6 ± 139458.0	0.038
	GLNUz	7.8 ± 1.7	9.2 ± 1.7	0.041
	ZLNU	12.5 ± 6.7	19.5 ± 12.2	N.S.
	ZP	0.240 ± 0.074	0.312 ± 0.101	N.S.
EBUS (n = 19)	FLR	4.12 ± 2.04	33.65 ± 40.79	0.013

**Abbreviations:** N.S. = not significant. GLCM = gray-level co-occurrence matrix. GLRLM = gray-level run length matrix. SRE = short-run emphasis. LRE = long-run emphasis. HGRE = high gray-level emphasis. SRHGE = short-run high gray-level emphasis. LRHGE = long-run high gray-level emphasis. GLNU = gray-level non-uniformity for run. RLNU = run length non-uniformity. RP = run percentage. NGLDM = neighborhood gray-level different matrix. GLZLM = gray-level zone length matrix. SZE = short-zone emphasis. LZE = long-zone emphasis. HGZE = high gray-level zone emphasis. SZHGE = short-zone high gray-level emphasis. LZHGE = long-zone high gray-level emphasis. GLNUz = gray-level non-uniformity for zone. ZLNU = zone length non-uniformity. ZP = zone percentage. EBUS = endobronchial ultrasound. FLR = fat-to-lesion strain ratio.

For the 19 patients who underwent EBUS elastography for FLR measurements, the mean FLR of the SCLC patients (n = 7, mean 33.7) was significantly higher than the mean FLR of the sarcoidosis patients (n = 12, mean 4.1) (P = 0.013). ROC curve analysis, with a cut-off value of 20.7, showed an AUROC of 0.857 and a sensitivity and specificity of 71% and 100%, respectively (Fig 4).

### Improvements in diagnostic capabilities of the combination of single CT texture features with other texture features or with EBUS elastographic FLR values

Multinomial logistic regression analysis was performed of combinations of representative texture features with other texture features or with FLR values. Overall, combinations of different texture features led to slight increases in the AUROCs. The AUROC of GLRLM-HGRE plus GLCM-contrast was 0.839, with a sensitivity and specificity of 86% and 88%, respectively. The AUROC of GLRLM-HGRE plus GLCM-dissimilarity was 0.844, with a sensitivity and specificity of 86% and 88%, respectively. Moreover, in 19 patients who underwent EBUS elastography for FLRs, the AUROC of GLRLM-HGRE plus FLR was 0.988, with a sensitivity and specificity of 100% and 92%, respectively. The cut-off values for discriminating sarcoidosis from SCLC were FLR > 2.83 and GLRLM-HGRE < 4780 for the combination. Among all the combinations we examined, the combination of GLRLM-HGRE plus FLR showed the highest diagnostic accuracy for differentiating sarcoidosis from SCLC (Fig 4).

## Discussion

This study first demonstrated that in patients with mediastinal lymphadenopathy on CT caused by SCLC metastasis or sarcoidosis, some texture features can discriminate SCLC from sarcoidosis. Further, the combination of the texture feature GLRLM-HGRE plus EBUS elastographic FLR achieved almost perfect diagnostic capability for differentiating between these 2 pathologies. We therefore believe that CT-based texture analysis of enlarged mediastinal lymph nodes might be useful for differentiating between SCLC and sarcoidosis, and might be useful for other diseases causing mediastinal lymphadenopathy, such as metastatic lymph nodes associated with non-small cell lung cancer (NSCLC) and lymph nodes affected by malignant lymphoma.

A recent study has demonstrated that complementary methods of texture analysis of mediastinal lymphadenopathy due to lung cancer on unenhanced CT images showed that the combination of GLCM with the experimental semivariogram feature produced an AUROC of 0.89, and a sensitivity, specificity, and accuracy of 75%, 70%, and 90%, respectively [7]. Also, CT texture features and shape analyses of mediastinal lymph nodes have shown a sensitivity, specificity, and accuracy of 81%, 80%, and 71%, respectively, in discriminating benign from malignant lesions [9]. Similar to these reports, in this study, the combination of 2 different texture features (GLRLM-HGRE plus GLCM-contrast or GLCM-dissimilarity) showed high diagnostic capability in discriminating between mediastinal lymphadenopathy caused by sarcoidosis and that caused by metastatic SCLC. Although speculating on the reasons is difficult why these GLRLM and GLCM features can differentiate between a lymphadenopathy caused by sarcoidosis and that caused by SCLC, differences between the histopathologic characteristics of the affected lymph nodes would affect the ability to discriminate. In general, GLRLM provides the size of homogeneous runs for each grey level, and GLCM is calculated from 13 different directions in a 3-dimensional model with a delta-voxel distance relationship between neighboring voxels. Lymph nodes affected by sarcoidosis had a higher value of GLRLM-HGRE than those affected by SCLC, which suggests that the density of the voxels in the ROIs on the CT scans was more homogeneous in lymph nodes affected by sarcoidosis than those affected by SCLC. Also, lymph nodes affected by SCLC showed higher GLCM-contrast and GLCM-dissimilarity values than lymph nodes affected by sarcoidosis, suggesting that density heterogeneity of lymph nodes affected by SCLC was greater than that in lymph nodes affected by sarcoidosis. These texture findings might reflect the histopathological characteristics of these 2 diseases. Compared with sarcoidosis, SCLC is histologically characterized by frequent and extensive necrosis [23], which may cause low-density clusters in the ROI of the lymph node on the CT image. Although we did not place an ROI on macroscopically necrotic areas of the mediastinal lymph nodes on CT images, the greater extent of heterogeneity in lymph nodes affected by SCLC that was implied by the CT-based texture analysis compared with the extent in lymph nodes affected by sarcoidosis, might reflect microscopic necroses in our patient cohort. This hypothesis should be investigated in future studies by comparing CT analyses with detailed histopathological findings.

Our study of FLRs obtained by elastography during EBUS-TBNA was an exploratory investigation. Elastography has been frequently used for the diagnosis of breast cancer, and the FLR has been reported to be useful for differentiating between benign and malignant breast lesions [16, 17]. Elastographic assessments of mediastinal lymph nodes have also been performed; it has been reported that EBUS elastography can be used an indicator for distinguishing between malignant and benign lymph nodes [11–14]. In this study, among the 19 patients who underwent EBUS elastography, the FLR values were significantly higher in those with SCLC than in those with sarcoidosis. Moreover, the combination of GLRLM-HGRE plus FLR showed an AUROC of 0.988, sensitivity of 100%, and specificity of 92%, which were the highest values in this study. This result suggests that the combination of texture analysis and FLR may be expanded to discriminating between mediastinal lymph nodes enlarged due to involvement with benign or malignant diseases. If future studies can demonstrate that the combination of CT-based texture analysis and FLR measurement by EBUS is truly useful for differentiating between mediastinal lymph nodes affected by benign versus malignant disease, the combination could be used to predict malignant involvement prior to TBNA and provide information for the selection of lymph nodes for sampling. CT-based texture analysis combined with EBUS-based FLR should enable a shorter examination time than the current EBUS-TBNA, thus reducing the need for agents used for sedation.

This study has several limitations. First, it only enrolled patients with SCLC and sarcoidosis. Other malignancies associated with mediastinal lymph node metastases such as NSCLC and lymphoma, were not included. Although the original purpose of this study was to investigate whether or not CT-based texture analysis can differentiate SCLC from sarcoidosis, it is currently unknown whether our observations could be applied to other malignant and benign diseases that cause mediastinal lymphadenopathy. Second, a small number of patients were enrolled in this study. Third, any other abnormalities in the lung field on chest CT were not assessed. In some patients, characteristic pulmonary findings that did not require texture analysis for diagnosis might have been found on the chest CT. Fourth, the FLR by EBUS was not measured in all patients. Since the AUROC curves of the FLR data were larger than the AUROC curves of representative CT-based texture features in this study, the FLR might be the best parameter for differentiating between sarcoidosis and SCLC. However, we also believe that any imaging information such as CT-based texture measurements obtained before bronchoscopy should be useful for the daily clinical care management of patients with mediastinal lymphadenopathy. Fifth, we did not evaluate the correlations between CT findings, including texture features, and detailed histopathological findings. In particular, if we had had detailed information on necrosis in the lymph nodes of SCLC patients, we would have been more confident about our observations, which might have also been more persuasive than our current results. Sixth, we did not include patients with malignant lymphoma in this study because very few patients with lymphoma underwent EBUS-TBNA. CT-based texture analysis of lymphadenopathy associated with lymphoma should be of interest for the daily clinical care of patients with mediastinal lymphadenopathy.

In conclusion, CT-based texture analysis is useful for discriminating between the mediastinal lymphadenopathy caused by sarcoidosis from that caused by metastasis from SCLC. When specific texture features were combined with EBUS elastographic FLR values, the diagnostic capabilities of the individual modalities for the differentiation between these 2 diseases was greatly improved.

## Supporting information

S1 Data (XLSX)
